# Murine Pancreatic Adenocarcinoma Reduces Ikaros Expression and Disrupts T Cell Homeostasis

**DOI:** 10.1371/journal.pone.0115546

**Published:** 2015-01-28

**Authors:** Nadine Nelson, Shengyan Xiang, Xiaohong Zhang, Danielle Gilvary, Julie Djeu, Kazim Husain, Mokenge Malafa, Nasreen Vohra, Shari Pilon-Thomas, Tomar Ghansah

**Affiliations:** 1 Department of Molecular Medicine, Morsani College of Medicine, University of South Florida, Tampa, FL, United States of America; 2 Department of Pathology and Cell Biology, Morsani College of Medicine, University of South Florida, Tampa, FL, United States of America; 3 Department of Immunology, H. Lee Moffitt Cancer Center, Tampa, FL, United States of America; 4 Department of Gastrointestinal Oncology, H. Lee Moffitt Cancer Center, Tampa, FL, United States of America; 5 Department of Surgery, Brody School of Medicine, East Carolina University, Greenville, NC, United States of America; Spanish National Cancer Centre (CNIO), SPAIN

## Abstract

**Background:**

Maintenance of T cell immune homeostasis is critical for adequate anti-tumor immunity. The transcription factor Ikaros is essential for lymphocyte development including T cells. Alterations in Ikaros expression occur in blood malignancies in humans and mice. In this study, we investigated the role of Ikaros in regulating T cell immune balance in pancreatic cancer mouse models.

**Methodology and Principal Findings:**

Using our Panc02 tumor-bearing (TB) mouse model, western blot analysis revealed a reduction in Ikaros proteins while qRT-PCR showed no differences in Ikaros mRNA levels in TB splenocytes compared to control. Treatment of naïve splenocytes with the proteasomal inhibitor, MG132, stabilized Ikaros expression and prevented Ikaros downregulation by Panc02 cells, *in vitro*. Western blot analyses showed a reduction in protein phosphatase 1 (PP1) and protein kinase CK2 expression in TB splenocytes while CK2 activity was increased. Immunofluorescence microscopy revealed altered punctate staining of Ikaros in TB splenocytes. Flow cytometry revealed a significant decrease in effector CD4^+^ and CD8^+^ T cell percentages but increased CD4^+^CD25^+^ regulatory T cells in TB splenocytes. Similar alterations in T cell percentages, as well as reduced Ikaros and CK2 but not PP1 expression, were observed in a transgenic, triple mutant (TrM) pancreatic cancer model. Ikaros expression was also reduced in enriched TB CD3^+^ T cells. MG132 treatment of naïve CD3^+^ T cells stabilized Ikaros expression in the presence of Panc02 cells. Western blots showed reduced PP1 and CK2 expression in TB CD3^+^ T cells.

**Conclusions/Significance:**

The results of this study suggest that the pancreatic tumor microenvironment may cause proteasomal degradation of Ikaros, possibly via dysregulation of PP1 and CK2 expression and activity, respectively. This loss of Ikaros expression may contribute to an imbalance in T cell percentages. Ikaros may potentially be a therapeutic target to restore T cell homeostasis in pancreatic cancer hosts, which may be critical for effective anti-tumor immunity.

## Introduction

Pancreatic ductal adenocarcinoma is currently the fourth leading cause of cancer-related deaths in the United States. Despite recent advances, successful treatment options against pancreatic cancer have had limited success due in part, to dampened anti-tumor immune responses that promote tumor progression [1,2]. Effector CD4^+^ and CD8^+^ T cells play important roles in the host’s anti-tumor immune responses as they facilitate destruction of tumor cells [3]. Regulatory T cells (Tregs) are a population of T cells that maintain peripheral immune tolerance against self-antigens and foreign antigens [1]. However, the critical balance between effector T cells and Tregs is lost in pancreatic cancer TB hosts, which may negatively impact anti-tumor immunity [4]. In particular, CD4^+^CD25^+^ Treg percentages are elevated in the peripheral blood of pancreatic cancer patients [5] as well as lymphoid organs in mice [6]. These elevated Treg numbers are associated with decreased CD8^+^ T cell percentages and lower survival rates [4]. Therefore, an imbalance in effector CD4^+^ and CD8^+^ T cells and regulatory T cells is a significant impediment to treating pancreatic cancer.

The Ikaros family of zinc finger transcription factors—Ikaros, Aiolos, Helios, Eos and Pegasus—play critical roles in hematopoiesis and lymphocyte development [7]. Ikaros, the founding member, encoded by the gene Izkf1, can activate and repress gene transcription and acts as a tumor suppressor in T cell lineages [8,9]. Mice expressing a non-DNA binding dominant-negative (DN) isoform of Ikaros exhibit severe defects including the absence of T cells after birth [10]. Additionally, mice with one disrupted and one functional copy of Ikaros display lymphocyte hyperproliferation and develop T-cell leukemias and lymphomas [8].

Ikaros is alternatively spliced, which produces functional and DN isoforms. The interaction of functional Ikaros isoforms with DN isoforms inhibit its activity [11]. Ikaros is also regulated by posttranslational modifications, which include phosphorylation [12,13]. Protein Kinase CK2 (formerly casein kinase 2) phosphorylation of Ikaros impairs its DNA binding ability, alters its subcellular localization and leads to its ubiquitin-mediated proteasomal degradation via phosphorylation in PEST regions (regions containing proline (P), glutamate (E), serine (S), and threonine (T) bordered by positively charged residues). In contrast, dephosphorylation of Ikaros by protein phosphatase 1 (PP1) maintains Ikaros stability and activity [14].

The necessity of Ikaros for normal lymphocyte development makes it a critical target to be examined in regulating immune responses in various diseases. Our study is one of the first to investigate Ikaros in pancreatic cancer, especially as it relates to effector and regulatory T cells. In this study, we provide evidence that loss of Ikaros expression occurs in pancreatic TB hosts. We show that this occurs, at least in part, by ubiquitin-mediated proteasomal degradation in response to pancreatic cancer factors. This protein degradation of Ikaros may be as a result of alterations in known regulators of its stability, PP1 and CK2. Loss of Ikaros expression may contribute to the observed imbalance in effector and regulatory T cell percentages, favoring an immunosuppressive microenvironment. Therefore, Ikaros may be a T-cell specific therapeutic target for maintaining T cell homeostasis in pancreatic cancer patients.

## Materials And Methods

### Cell Culture

The murine pancreatic adenocarcinoma Panc02 cell line was established by Corbett et al. [15]. This cell line was maintained in RPMI 1640 medium supplemented with 10% fetal bovine serum (FBS), (HyClone), 2 mM L-glutamine, 100 U/ml penicillin, 100 μg/ml streptomycin (Gibco) at 37°C in 5%CO2. Cultured cells were tested and found to be negative for mycoplasma and viral contamination [16].

### Mice

Female C57BL/6 mice (6–8 weeks) were purchased from Harlan Laboratories (Indianapolis). The Institutional Animal Care and Use Committee of the University of South Florida approved protocol R4152 in compliance with the Guide for the Care and Use of Laboratory Animals. The mice were maintained in a pathogen-free animal facility for 1 week before the start of experiments. Mice were subcutaneously (s.c.) injected with 1.5×10^5^ murine Panc02 cells (TB) or 100 μl PBS (control) on the lower, ventral abdomen. Every three days, the mice were weighed and tumors were measured using a digital caliper [16]. Spleens from transgenic LSL-Kras^G12D/+;^LSL-Trp53^R172H/+^;Pdx-1-Cre mice, known as triple mutant (TrM) mice, were also used in this study [17]. Mice were euthanized using CO_2_ and cervical dislocation according to the University of South Florida IACUC guidelines.

### Western Blotting

Protein lysates were prepared from splenocytes of control, TB and TrM mice and *in vitro* treated naïve splenocytes using modified Radioimmunoprecipitation assay (RIPA) Buffer (Millipore) supplemented with Na_3_OV_4_ and protease inhibitor cocktail (Sigma-Aldrich). Protein concentrations were determined using the BCA Protein Assay Kit (Thermo Fisher Scientific). A maximum of 40 μg cell protein lysates were loaded and resolved using NuPAGE 4–12% Bis-Tris polyacrylamide Gels (Invitrogen) or 12% hand cast gels and transferred to nitrocellulose membranes (Whatman). The membranes were blocked with 5% nonfat milk in PBS/0.1% Tween-20 and then probed with anti-Ikaros (Cell Signaling), at a dilution of 1:1000, anti-p53 (Santa Cruz), anti-CK2α (Santa Cruz Biotechnology) and anti-PP1 (Santa Cruz Biotechnology) at a dilution of 1:200. Primary antibodies were detected using their respective secondary IgG, HRP-conjugated antibodies (Jackson Immunoresearch), at a dilution of 1:10000. Secondary antibodies were identified using Super Signal West Pico and Femto Chemiluminescent Substrates (Thermo Fisher Scientific). As an internal control for equal protein loading, all blots were stripped and re-probed with anti-ß-actin (Sigma-Aldrich) at a dilution of 1:20,000 or anti-GAPDH (Santa Cruz Biotechnology) at a dilution of 1:200. Membranes were either exposed to x-ray films (Phoenix) and developed using a Kodak M35-X OMAT Processor or imaged using a ChemiDoc XRS Imaging System (Bio-Rad). Band intensities were quantified using Quantity One 1-D densitometry and Image Lab softwares (Bio-Rad) [16].

### Quantitative RT-PCR (qRT-PCR)

Total RNA was extracted from single-cell suspensions of control and TB whole splenocytes using TRI Reagent (Molecular Research Center). cDNA was then synthesized using the High Capacity cDNA Reverse Transcription Kit (Applied Biosystems). Ikaros mRNA expression was detected by qRT-PCR using SYBR Green JumpStart Taq Ready Mix (Sigma-Aldrich) and an AB StepOne Plus Real-Time PCR System under the following conditions: 95°C for 10 min followed by 40 cycles of 95°C for 15 sec and 60°C for 1 min, and primers: forward, 5′-CAT AAA GAG CGA TGC CAC AA-3′, reverse, 5′-CAG GAC AAG GGA CCT CTC TG-3′ [18]. Each sample was assayed in triplicate. GAPDH was amplified as the internal control and reference gene. Normalization to GAPDH was used to determine relative mRNA frequency using the Comparative CT method [16].

### In vitro Assays

Single-cell suspensions of whole and CD3^+^ enriched T cells from splenocytes from naïve mice were cultured in the presence or absence of murine Panc02 cells and/or the proteasome inhibitor (carbobenzoxyl-L-leucyl-L-leucyl-L-leucine) Cbz-LLL (MG132; Sigma-Aldrich) at the indicated concentrations for four hours *in vitro*. Protein lysates of *in vitro* treated-splenocytes were prepared and analyzed for Ikaros protein expression using western blot analysis.

### In vitro CK2 Kinase Assay

CK2 kinase activity was measured using the CK2 assay kit (Millipore) according to the manufacturer’s instructions. CK2 activity was calculated by subtracting the mean counts per minute (CPM) of samples in the absence of substrate from the mean CPM of samples in the presence of the substrate.

### Immunofluorescence Microscopy

Cytospin slides of control and TB splenocytes were prepared and fixed at −20°C in methanol:acetone (3:1). These cells were then stained with a rabbit polyclonal against Ikaros (Santa Cruz Biotechnology) diluted 1:200 in 0.1% Nonidet P-40 in 1% BSA in PBS for 1 h. Slides were washed and incubated with a secondary goat anti-rabbit Alexa Fluora 594 antibody (Life Technologies) diluted 1:200 in 0.1% Nonidet P-40 in 1% BSA in PBS for 30 mins. Appropriate isotype controls were used to check for non-specific binding which was not detected. Slides were washed in PBS and cover slips were applied and mounted using ProLong Gold Antifade Mountant with DAPI (Life Technologies). Immunofluorescence was imaged using a Zeiss Olympic Microscope and analyzed using Image J Software [19].

### Flow Cytometry

Splenocytes were harvested from control, TB and TrM mice and single-cell suspensions were made using a cell dissociation sieve (Sigma-Aldrich) and 70 μm cell strainers (BD Falcon). Red blood cells (RBC) were lysed using RBC lysis buffer (eBioscience). Cells were then suspended in 3%FBS-PBS and stained with antibodies against T cell surface markers CD3 (FITC) (eBioscience), CD4 (Pe-Cy7) (BD Pharmingen), CD8 (APC-H7) (BD Pharmingen) and CD25 (PE) (eBioscience). Flow Cytometry was performed using a BD LSRII (BD Biosciences Immunocytometry Systems) and data analyzed with FlowJo software (Tree Star Inc.) [16].

### CD3^+^ T Cell Enrichment

Whole splenocytes from control and TB mice were processed into single-cell suspensions, as previously described. CD3^+^ T cells were purified (~90% purity) from whole splenocytes by positive selection using anti-CD3-1-PE and anti-PE-magnetic microbeads on an AutoMACS Pro Separator [6,16] or using the EasySep Mouse T Cell Enrichment kit according to the manufacturer’s protocol (Stem Cell Technologies).

### Statistical Analysis

All *in vivo* and *in vitro* results described in this study are representative of the mean ± S.E.M. of at least three independent experiments analyzed with two-tailed Student’s t test using PRISM 5 software (GraphPad, San Diego, CA). Differences were considered significant at p<0.05.

## Results

### Reduced Ikaros expression in TB mice

**Fig 1:**
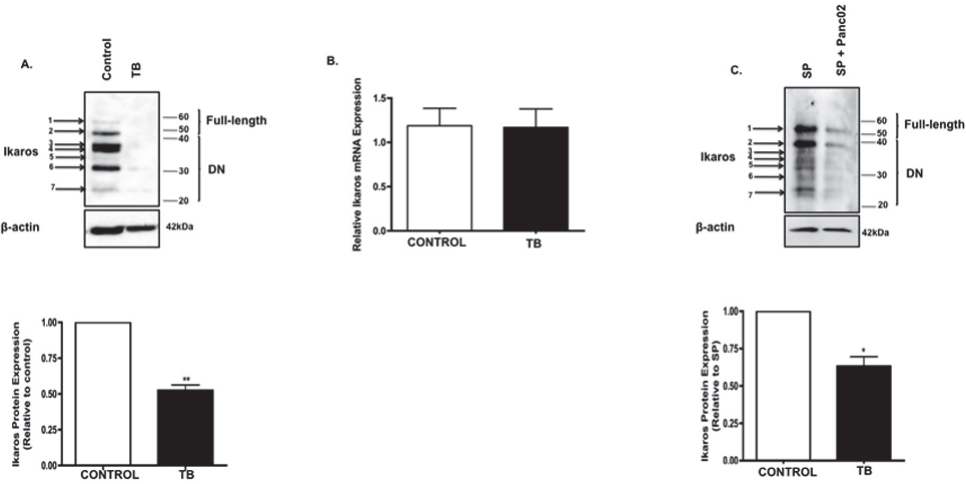
Reduced Ikaros expression in TB mice. **A**. Western blot analysis of Ikaros protein expression in control and TB splenocytes. To control for equal protein loading the blot was reprobed with an antibody specific to β-actin. The arrows on the left indicate observed Ikaros isoforms. Representative quantification of normalized densitometric ratios of western blot data is shown. **B**. qRT-PCR analysis of Ikaros mRNA expression in control and TB mice. **C**. Western blot analysis of Ikaros protein expression in naïve splenocytes co-cultured with Panc02 cells. To control for equal protein loading the blot was reprobed with an antibody specific to β-actin. The arrows on the left indicate observed Ikaros isoforms. Representative quantification of normalized densitometric ratios of western blot data is shown. Represented is the mean ± S.E.M. of control (n = 3) compared to TB (n = 3) mice.**p<0.005 (by two-tailed Student’s t test).

Ikaros is a critical regulator of lymphocyte development and is characterized as a tumor suppressor gene [20]. More specifically, loss of Ikaros activity due to genetic or functional inactivation leads to the development of leukemias and lymphomas in mice and humans [8,21,22]. However, investigations into the role of Ikaros in solid cancers, especially as it relates to immune cell development, have been limited. We therefore wanted to determine whether defects in Ikaros might occur in a pancreatic tumor microenvironment. Ikaros protein expression was detected in the peripheral blood, bone marrow (data not shown) and spleen of our TB mice. However, its expression was most abundant in the spleen, which was used in this study. We first evaluated Ikaros protein expression in splenocytes from control and TB mice by using an antibody to the conserved C-terminus to detect all possible isoforms expressed. Western blot analyses revealed the expression of at least 7 Ikaros isoforms in control splenocytes which, based on their molecular weight (MW), appear to correspond to full-length isoforms Ik-1, and Ik-2/3 (arrows 1 and 2; **Fig. 1A**) and five smaller (<46), DN isoforms [23,24] (arrows 3–7; **Fig. 1A**). Expression of these isoforms was downregulated in TB splenocytes and accounted for a significant (two-fold) decrease in total Ikaros protein expression in TB splenocytes compared to control (**Fig. 1A**). Next, we evaluated mRNA expression of Ikaros in control and TB mice to determine whether differences in Ikaros protein expression were due to changes in its transcript. Using primers that detect Ikaros isoforms through conserved regions, we found no significant difference in total Ikaros mRNA expression between TB and control splenocytes (**Fig. 1B**). Observing reduced Ikaros protein expression in TB mice, we then investigated whether this downregulation was in response to Panc02 factors (soluble and non-soluble). We recapitulated the *in vivo* tumor microenvironment by co-culturing splenocytes from naïve C57BL/6 mice with murine Panc02 cells *in vitro*. This co-culture resulted in reduced Ikaros protein expression in splenocytes as revealed by western blot analysis (**Fig. 1C**). Thus far, these results suggest that pancreatic cancer factors may downregulate Ikaros expression in TB mice.

### Murine Panc02 cells cause ubiquitin-mediated proteasomal degradation of Ikaros *in vitro*

**Fig 2:**
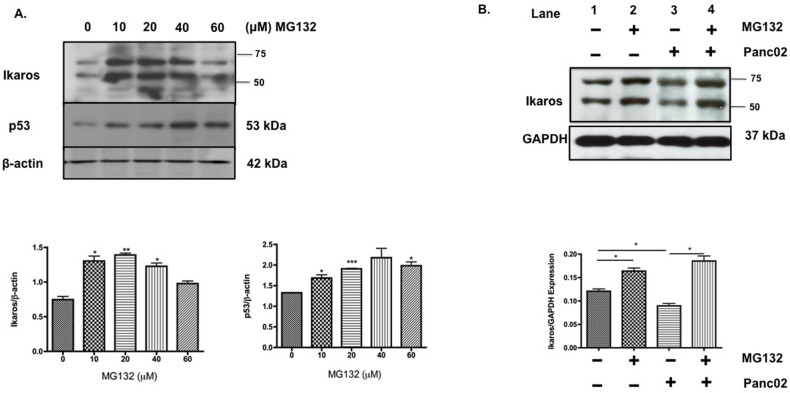
Murine Panc02 cells cause ubiquitin-mediated proteasomal degradation of Ikaros *in vitro*. **A**. Western blot analysis of Ikaros and p53 expression in naïve splenocytes treated with the proteasomal inhibitor, MG132 for four hours *in vitro*. To control for equal protein loading the blot was reprobed with an antibody specific to β-actin. Representative quantification of normalized densitometric ratios of western blot data is shown. **B**. Western blot analysis of Ikaros expression in naïve splenocytes co-cultured in the absence or presence of Panc02 cells and/or MG132. To control for equal protein loading the blot was reprobed with an antibody specific to GAPDH. Representative quantification of normalized densitometric ratios of western blot data is shown. Represented is the mean ± S.E.M. of three independent experiments. *p<0.05, **p<0.005; ***p<0.0001(by two-tailed Student’s t test).

Our data proposes that downregulation of Ikaros protein expression in TB splenocytes may be due to a posttranslational modification affecting its protein stability. Studies have shown that Ikaros protein undergoes ubiquitin-proteasomal degradation [14,25–28]. As Ikaros expression is significantly reduced in TB splenocytes, we treated naïve splenocytes with the proteasomal inhibitor, MG132, which was used as a molecular tool to test whether Ikaros protein undergoes proteasomal degradation. Results showed that in the presence of MG132, particularly at 10μM, 20μM and 40μM, there was a significant increase in Ikaros protein expression (**Fig. 2A**). MG132 inhibition of the proteasome blocks apoptosis and stabilizes p53 expression [29]. We therefore evaluated p53 expression to confirm MG132 activity in these experiments (**Fig. 2A**). Furthermore, we wanted to determine whether the downregulation of Ikaros in TB mice was as a result of proteasomal degradation of Ikaros in response to Panc02 factors. Results of western blot analyses of splenocytes co-cultured with Panc02 cells showed that 10μM MG132 stabilized Ikaros expression (lane 2 vs. lane 1; **Fig. 2B**). However, in the presence of Panc02 cells Ikaros protein expression was reduced in splenocytes (lane 3 vs. lane 1; **Fig. 2B**). Interestingly, the addition of MG132 to the co-culture prevented Panc02-induced downregulation of Ikaros expression (lane 4 vs. lane 3; **Fig. 2B**). These data suggest that pancreatic cancer factors may cause downregulation of Ikaros via protein degradation by the ubiquitin-proteasome pathway.

### Altered PP1 expression, CK2 activity and Ikaros nuclear staining pattern in TB mice

**Fig 3:**
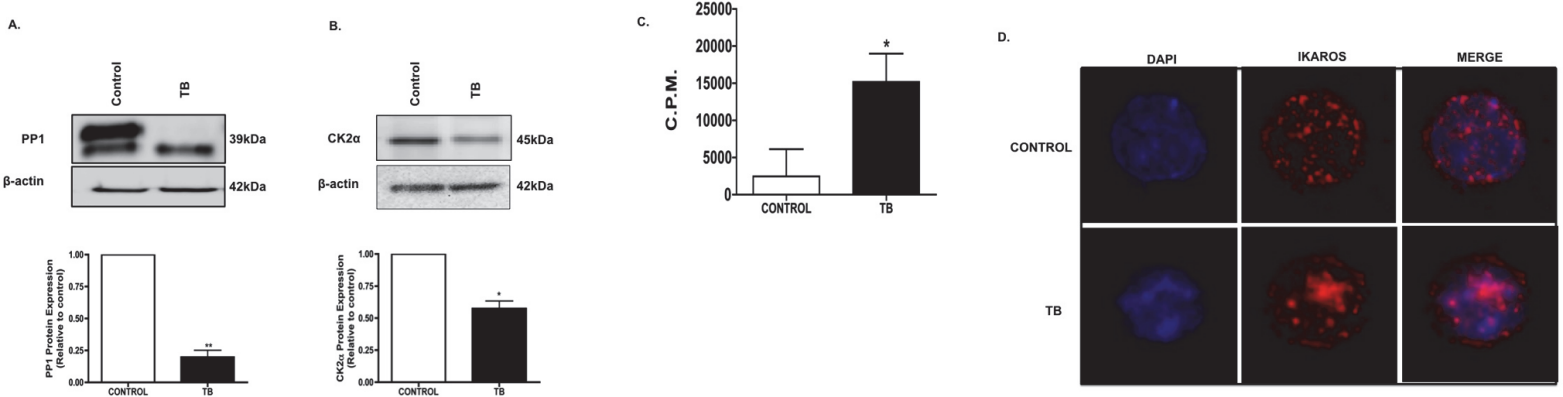
Altered PP1 expression, CK2 activity and Ikaros nuclear staining pattern in TB mice. Western blot analysis of **A**. PP1 and **B**. CK2α protein expression in control and TB splenocytes. To control for equal protein loading the blots were reprobed with an antibody specific to β-actin. Representative quantifications of normalized densitometric ratios of each western blot are shown. **C**. Counts per minute (C.P.M.) of CK2 activity in protein lysates from splenocytes from control and TB mice as assayed by an *in vitro* CK2 kinase assay. Represented is the mean ± S.E.M. of control (n = 3 compared to TB (n = 3) mice).*p<0.05, **p<0.005; (by two-tailed Student’s t test). **D**. Immunofluorescence microscopy showing Ikaros expression and subcellular localization in control and TB splenocytes (n = 25 cells). Nuclear DNA appears as blue (DAPI), Ikaros as red (Ikaros panel) and Ikaros and DAPI combined (Merged panel). (Magnification, ×240). Representative results from at least three independent immunofluorescence microscopy experiments.

A balance between CK2 and PP1 is responsible for maintaining Ikaros’ protein stability and function. In particular, lack of dephosphorylation by PP1 and hyperphosphorylation by CK2 leads to increased degradation of Ikaros [13,14,30]. Since our data suggests that Ikaros downregulation may be as a result of its protein degradation, we investigated the expression of CK2 and PP1 in splenocytes from our control and TB mice. We firstly evaluated PP1 expression by western blot analyses using an antibody that recognizes PP1 catalytic subunits. Western blot analsyes detected two catalytic isoforms in control splenocytes. However, the higher MW PP1 catalytic isoform was reduced in TB splenocytes (**Fig. 3A**). Next, we evaluated CK2 by also evlauting the expression of its catalytic subunit. We found a reduction in CK2α protein expression in TB splencoytes compared to control (**Fig. 3B**). We also assayed CK2 activity which revealed a significant increase in CK2 activity in TB splenocytes compared to control (**Fig. 3C**). Phosphorylation/dephosphorylation of Ikaros by CK2 and PP1 also affects its DNA binding ability and subcellular localization. The majority of Ikaros localizes at pericentromeric heterochromatin (PC-HC) where it functions in regulating gene expression [14]. Therefore, having observed defects in PP1 and CK2 pathways, we evaluated Ikaros localization using immunofluorescence microscopy. In control splenocytes, we observed the characterisitic nuclear, punctate staining pattern of Ikaros, indicative of its PC-HC localization (**Fig. 3D**). However, in TB splenocytes, more diffuse staining of Ikaros was observed (**Fig. 3D**). Overall, we observed reduced PP1 expression and increased CK2 activity as well as cytoplasmic subcellular localization of Ikaros in TB splenocytes. Therefore, differential expression of these two critical proteins may contribute to regulating Ikaros expression in our TB mice.

### Altered T cell percentages in TB and TrM mice

**Fig 4:**
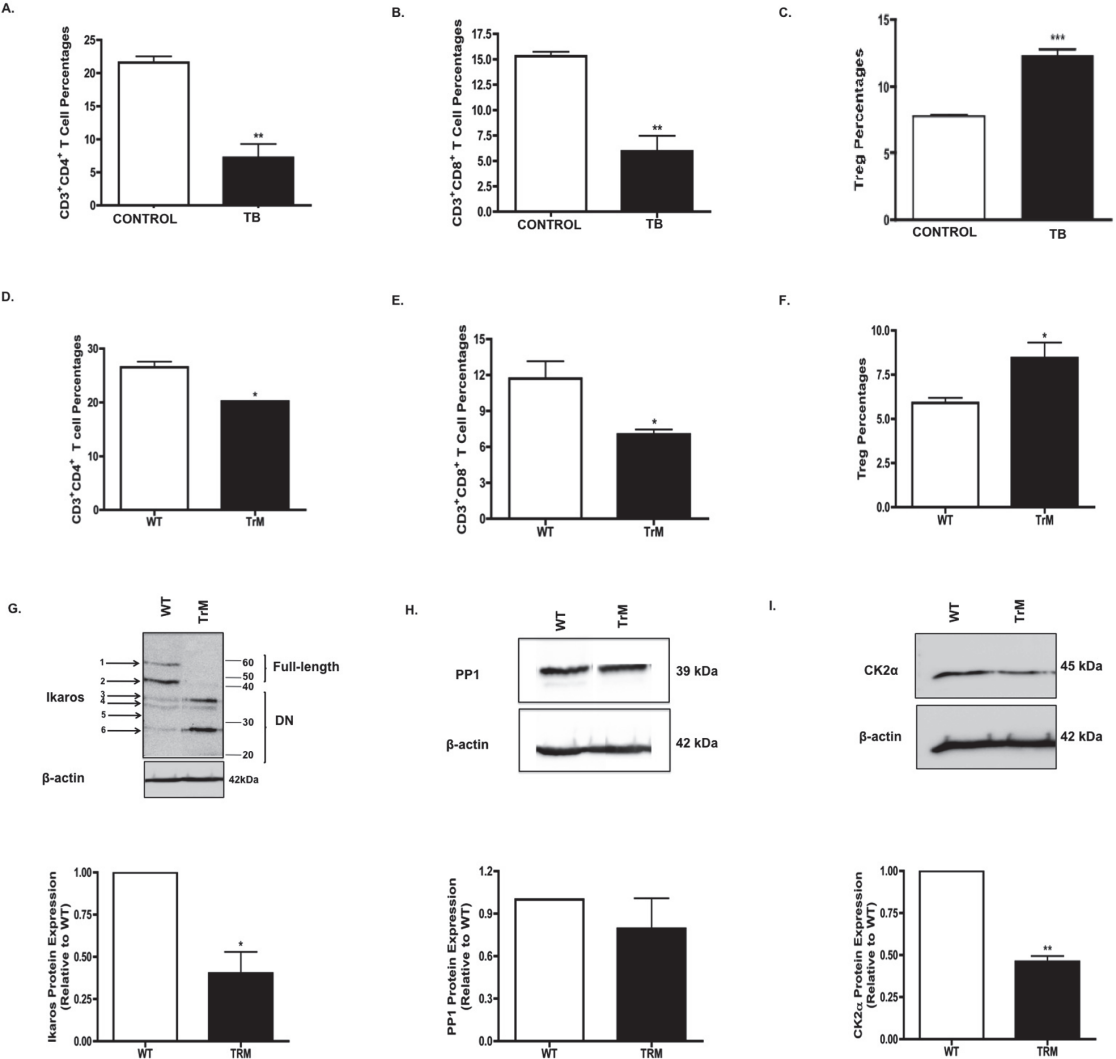
Altered T Cell Percentages in TB and TrM mice. Flow cytometry analysis of T cell percentages in TB and TrM mice. **A**. CD4^+^ T cell, **B**. CD8^+^ T cell and **C**. Treg percentages in splenocytes from control and TB mice. **D**. CD4^+^ T cell, **E**. CD8^+^ T cell and **F**. Treg percentages in splenocytes from wild-type (WT) and TrM mice. Western blot analysis of **G**. Ikaros **H**. PP1 and **I**. CK2α protein expression in WT and TrM splenocytes. To control for equal protein loading the blot was reprobed with an antibody specific to β-actin. The arrows on the left indicate observed Ikaros isoforms. Representative quantifications of normalized densitometric ratios of western blot data are shown. Represented is the mean ± S.E.M. of control (n = 3 compared to TB (n = 3) mice).*p<0.05;**p<0.005; ***p<0.0001(by two-tailed Student’s t test).

Ikaros has been identified as a regulator of T cell development [8,31,32]. T cells, specifically CD4^+^ and CD8^+^ T cells, are key players in tumor protective immunity [33,34]. Having observed defects in Ikaros expression, we next evaluated whether T cell development is altered in response to murine pancreatic cancer. Flow cytometry results showed that there was a significant decrease in both CD4^+^ (**Fig. 4A**) and CD8^+^ (**Fig. 4B**) effector T cell percentages in splenocytes from TB compared to control mice. Given the reduction in effector T cell percentages in splenocytes from TB mice, we investigated the percentages of immunosuppressive regulatory T cells. Flow cytometry results showed that there was a significant increase in CD4^+^CD25^+^ Tregs in splenocytes from TB compared to control mice (**Fig. 4C**). We previously published that CD4^+^CD25^+^ Tregs from TB mice suppress antigen-specific CD8^+^ T cell responses in a dose dependent manner, at a greater rate as compared to control Tregs [6]. Thus far, our results suggest that defects in Ikaros expression may be associated with a loss of T cell equilibrium in our pancreatic TB mice. Our next step was to determine whether this disruption in effector and regulatory T cell balance occurred in another highly translatable, transgenic mouse model of pancreatic cancer. The LSL-Kras^G12D/+;^LSL-Trp53^R172H/+^;Pdx-1-Cre transgenic mouse model (TrM mice) has mutations in Kras and p53, leading to spontaneous development of pancreatic cancer [17], that recapitulates pancreatic cancer in humans [35]. Flow cytometry analyses showed a reduction in effector CD4^+^ (**Fig. 4D**) and CD8^+^ T (**Fig. 4E**) cells but an increase in regulatory T cells (**Fig. 4F**) in splenocytes from TrM mice compared to wild-type (WT) littermates. We then evaluated the expression of Ikaros, CK2 and PP1 in these TrM mice to delineate their possible involvement in regulating T cell immune homeostasis in this model. Western blot analyses revealed a significant reduction in overall Ikaros expression in splenocytes of triple mutant mice compared to wild-type (WT) mice (**Fig. 4G**). However, in the TrM splenocytes, Ikaros DN isoforms were mainly expressed (**Fig. 4G**). There was no significant difference in protein expression of PP1 catalytic subunits (**Fig. 4H**) but CK2α expression was reduced (**Fig. 4I**) in TrM compared to WT mice. This implies that Ikaros dysregulation, the possible involvement of PP1 and/or CK2, and the resulting imbalance in T cell profiles, may have clinical relevance in pancreatic cancer.

### Dysregulation of Ikaros, PP1 and CK2 in CD3^+^ enriched T cells

**Fig 5:**
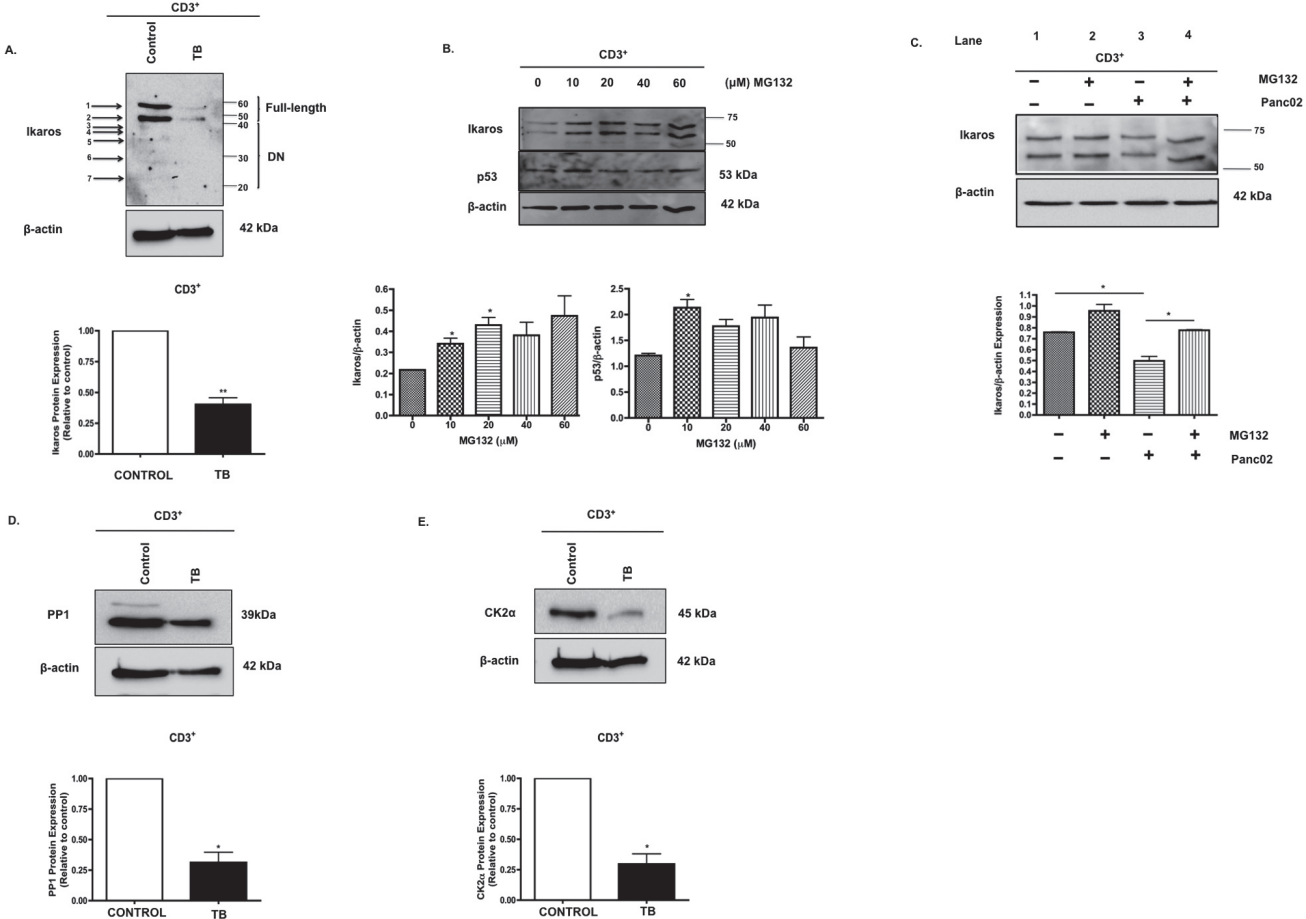
Dysregulation of Ikaros, PP1 and CK2 in CD3^+^ enriched T cells. **A**. Western blot analysis of Ikaros protein expression in control and TB CD3^+^ T cells. To control for equal protein loading the blot was reprobed with an antibody specific to β-actin. The arrows on the left indicate observed Ikaros isoforms. Representative quantification of normalized densitometric ratios of western blot data is shown. **B**. Western blot analysis of Ikaros and p53 expression in naïve CD3^+^ T cells treated with the proteasomal inhibitor, MG132 for four hours *in vitro*. To control for equal protein loading the blot was reprobed with an antibody specific to β-actin. Representative quantification of normalized densitometric ratios of western blot data is shown. **B**. Western blot analysis of Ikaros expression in naïve CD3^+^ T cells co-cultured in the absence or presence of Panc02 cells and/or MG132. To control for equal protein loading the blot was reprobed with an antibody specific to GAPDH. Western blot analysis of **D**. PP1 and **E**. CK2α protein expression in control and TB CD3^+^ T cells. To control for equal protein loading the blots were reprobed with an antibody specific to β-actin. Representative quantifications of normalized densitometric ratios of western blot data are shown. Represented is the mean ± S.E.M. of control (n = 3 compared to TB (n = 3) mice).*p<0.05, **p<0.005; (by two-tailed Student’s t test).

Thus far, we have observed a loss of Ikaros expression and T cell homeostasis in whole splenocytes from TB compared to control mice. Reports show that modulation of Ikaros expression in T cells affects their polarization, proliferation and differentiation [31,36]. Next, we investigated whether Ikaros expression was specifically altered at the T cell level in our animal model and could account for the loss of T cell homeostasis observed. Correlating with results in whole splenocytes, western blot analysis showed that Ikaros protein expression was also significantly reduced in enriched CD3^+^ T cells from TB mice compared to control (**Fig. 5A**). The isoforms detected appear to correlate with Ik-1 and Ik-2/3, which have previously been reported to be predominantly expressed in T lymphocytes [13]. We then evaluated whether Ikaros expression in T cells is also regulated by ubiquitin-mediated proteasomal degradation. CD3^+^ T cells enriched from naïve splenocytes were treated with increasing concentrations of MG132 *in vitro* as previously described. Western blot analyses of Ikaros expression revealed that MG132 did in fact significantly increase Ikaros expression in CD3^+^ T cells at 10 and 20μM concentrations with MG132 activity evaluated by p53 expression (**Fig. 5B**). Next, we co-cultured these enriched CD3^+^ T cells with Panc02 cells in the absence or presence of MG132. MG132 stabilized Ikaros expression in T cells (lane 2 vs. lane 1; **Fig. 5C**). Panc02 cells caused reduced Ikaros expression in T cells (lane 3 vs. lane 1; **Fig. 5C**). However, this downregulation was prevented in the presence of 10μM MG132 (lane 4 vs. lane 3; **Fig. 5C**), suggesting that Panc02 factors contribute to proteasomal degradation of Ikaros in CD3^+^ T cells. Our next step was to determine the expression of PP1 and CK2, regulators of Ikaros, in these isolated CD3^+^ T cells. Similar to our results in whole splenocytes, there was a reduction in PP1, especially of the higher MW catalytic isoform in TB CD3^+^ T cells compared to control (**Fig. 5D**). There was also a significant reduction in CK2α expression in TB CD3^+^ T cells compared to control (**Fig. 5E**). These data indicate that dysregulation of Ikaros in CD3^+^ T cells, possibly as a result of altered in PP1 and CK2 expression and activity, may contribute to loss of T cell homeostasis in pancreatic TB mice.

## Discussion

**Fig 6:**
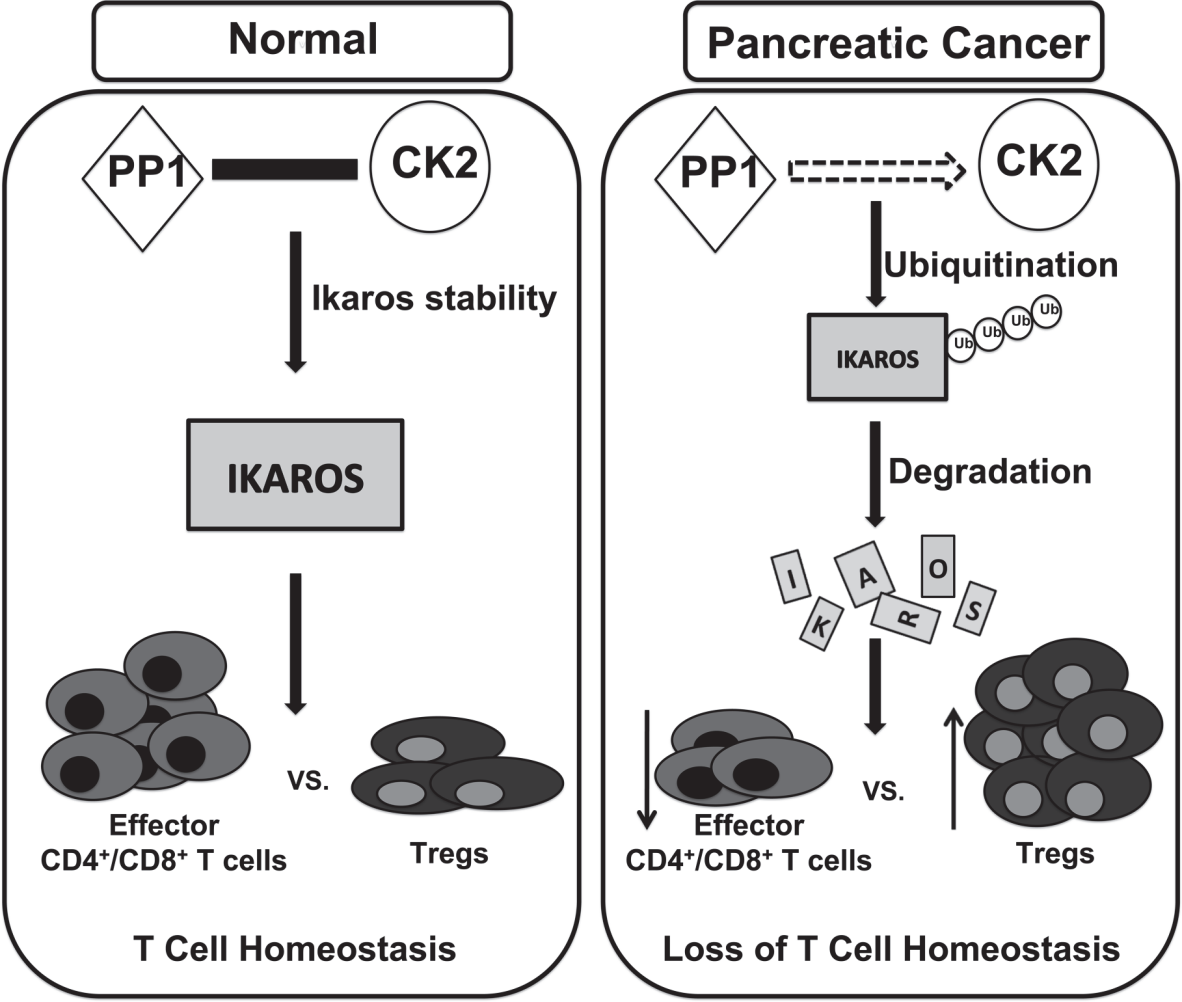
Proposed Model. Murine pancreatic cancer causes Ikaros degradation and alters T Cell Homeostasis. We propose a potential molecular mechanism of Ikaros regulation by which under normal conditions, the balance (represented by the solid, black bar) in the concerted action of PP1 and CK2 stabilizes Ikaros protein expression. This results in maintenance of effector CD4/CD8^+^ and regulatory T cell percentages. However, in a pancreatic cancer microenvironment, our findings suggest that there is a decrease in PP1 but an increase in CK2 activity (represented by broken arrow), which leads to ubiquitin-mediated protein degradation of Ikaros. This loss of Ikaros expression results in a loss of T cell homeostasis marked by a reduction of effector CD4/CD8^+^ T cell percentages and an increase of regulatory T cells. Ikaros may therefore be important for regulating T cell immune responses in pancreatic cancer.

Ikaros is a critical regulator of lymphocyte development, especially T cells. In fact, Ikaros has been proposed to function as a tumor suppressor in hematological malignancies [37–39]. However, the role of Ikaros in solid cancers has not been fully investigated. In this study, we identified the possible involvement of Ikaros in T cell homeostasis in pancreatic cancer mouse models. Our results suggest that pancreatic cancer (soluble and non-soluble) factors cause a reduction in Ikaros expression in splenocytes. We provide evidence that suggests these pancreatic cancer factors cause ubiquitin-mediated proteasomal degradation of Ikaros, which may be as a result of dysregulation in PP1 and CK2 pathways. Furthermore, we showed that this loss of Ikaros coincides with an imbalance in T cell immune responses resulting in decreased percentages of effector CD4^+^ and CD8^+^ T cells and increased regulatory T cell percentages (**Fig. 6**). Our study therefore proposes a putative and novel role for Ikaros in regulating T cell homeostasis in pancreatic cancer hosts.

The Ikaros gene is alternatively spliced to generate multiple full-length DNA binding and DN isoforms [21]. We observed at least 7 splice variants of Ikaros are expressed in control splenocytes, all of which are downregulated in TB mice. TrM LSL-Kras^G12D/+;^LSL-Trp53^R172H/+^;Pdx-1-Cre transgenic mice have mutations in p53 and Kras that model the genetic diversity of humans with pancreatic cancer, making it a highly translatable model. In WT littermates for TrM mice, at least 6 isoforms were observed. We are in the process of identifying these specific isoforms and comparing them to the currently identified isoforms and their variants [24]. Ik-1 and Ik-2/3, are reduced in our TB and TrM models, which both have defects in T cell immune balance. These isoforms are amongst the predominant isoforms generally expressed in T cells [13]. Interestingly, these isoforms are also reduced in our CD3^+^ TB T cells and are the main isoforms undergoing proteasomal degradation. Therefore, these full-length Ikaros isoforms may be critical for maintaining T cell immune balance, while the overexpression of DN isoforms may cause an imbalance and needs to be further investigated. In addition, we are also investing the alternative splicing mechanisms that may govern Ikaros isoform expression in our pancreatic cancer models. Defects in Ikaros expression as well as the shift in T cell immune balance in the spontaneous pancreatic cancer mice, provide evidence that Ikaros may indeed have clinical relevance in regulating effector and regulatory T cell immune responses in pancreatic cancer hosts.

The reduction in protein but not mRNA expression of Ikaros in TB mice led us to believe that Ikaros protein may be regulated posttranslationally. Initial studies showed that Ikaros is subject to protein degradation via the ubiquitin-proteasome pathway [14] and eliminated the involvement of proteolysis by calpains [25]. The ability of MG132 to increase Ikaros expression in both whole and CD3^+^ T cell enriched splenocytes provided evidence that the ubiquitin proteasome pathway may regulate Ikaros expression in immune cells in our pancreatic cancer mouse model. A number of recent studies support our findings as they have shown that Ikaros is indeed subject to proteasomal degradation [27,28], especially as it relates to T cells [26]. Our study further suggests that pancreatic cancer factors may trigger this proteasomal degradation of Ikaros. We published that Panc02 cells produce a number of inflammatory factors [16] and are investigating the molecular mechanism(s) by which inflammatory factors may modulate Ikaros’ expression in our pancreatic cancer models.

We also aimed to determine the pathway(s) involved in regulating Ikaros degradation in our TB mice. The concerted action of CK2 and PP1, controls Ikaros’ stability, DNA binding ability and subcellular localization [14]. Hyperphosphorylation by CK2 induces Ikaros’ degradation while dephosphorylation of Ikaros by PP1 increases its stability [14]. Having observed that downregulation of Ikaros protein may be as a result of ubiquitin-mediated proteasomal degradation, we hypothesized that this mechanism may be due to increased CK2 vs. PP1 activity in TB mice. We observe a reduction in PP1, specifically of a particular catalytic isoform, which is currently being investigated for its possible role in regulating Ikaros expression in TB mice. We also detected a reduction in CK2α expression but increased CK2 activity in TB splenocytes. In our TrM model, the lower MW catalytic subunit of PP1 was expressed and there was no difference in its expression compared to WT littermate controls. However, CK2α expression was also downregulated in these TrM mice but its activity has not yet been assayed. We are investigating how CK2 activity is regulated in our models. We have preliminary evidence that suggest that use of a selective CK2 inhibitor, increases Ikaros expression *in vitro* and *in vivo*, and restores effector and regulatory T cell balance in TB mice compared to control (unpublished data). This data further suggests that CK2 may be regulating Ikaros expression and function in our pancreatic cancer model. Currently, experiments using specific CK2 and PP1 inhibitors are being performed to confirm the roles of these two proteins in regulating Ikaros expression in pancreatic TB mice.

Defects in PP1 and CK2 can also affect Ikaros’ function in binding DNA and its subcellular localization [14]. In control splenocytes, Ikaros may be functional as its normal nuclear punctate staining was observed, which is characteristic of Ikaros localization to PC-HC which is essential for DNA binding and dimerization abilities [40]. The diffuse, nuclear staining pattern suggests that Ikaros may not be localized to PC-HC and may therefore be functionally inactive in TB mice. In fact, this phenotype is similar to that observed in Ikaros mutants unable to interact with PP1 [14]. It is also common in leukemic cells from infants with newly diagnosed ALL in which DN isoforms are prevalent [41], further supporting our findings.

We have shown that defects in Ikaros expression appear to limit the normal balance of T lymphocytes in our pancreatic cancer models. However, we have not specifically identified which T cell subsets mentioned (CD4^+^, CD8^+^, regulatory T cells) have intrinsic defects in Ikaros. Other CD3^+^ T cell populations such as T helper (Th) cells (Th1, Th2, Th9, Th17), induced regulatory T cells (iTregs), natural regulatory T cells (nTregs), natural killer T (NK/T) cells, and CD8^+^ regulatory T cells, etc. may also be regulated by Ikaros and could potentially be included in our results. We have observed defects in some of these T cell populations in our TB mice and are attempting to analyze these populations individually to identify possible defects in Ikaros expression, regulation and function. This will also allow us to identify other possible mechanisms by which Ikaros may be regulating T cell homeostasis in our model. These mechanisms may involve dysregulation of essential transcription factors [42,43] and cytokines [44,45] as well as alterations in cellular processes such as polarization, differentiation, proliferation [31,36], anergy [46] and apoptosis [25] as reported in other studies. Helios, another Ikaros family member, has been reported to complex with Ikaros in T cells and may limit Ikaros function [47]. Helios also plays a role in T cell activation and proliferation [48] and is involved in regulatory T cell development and function [49,50]. We have generated preliminary evidence that shows that Helios expression is downregulated in our TB mice (unpublished data) and are currently investigating its involvement along with Ikaros. We are also investigating Eos and Aiolos, other Ikaros family members, due to loss of homeostasis of other lymphocyte populations in this murine pancreatic cancer model (unpublished data).

This study is one of the firsts to investigate the possible involvement of Ikaros in regulating T cell immune homeostasis in pancreatic cancer. Our results show that pancreatic cancer factors cause reduced Ikaros expression in splenocytes, which may be as a result of Ikaros protein degradation by the ubiquitin/proteasome pathway. We also provide evidence that activation of this pathway may involve dysregulation of the balance between PP1 phosphatase and CK2 kinase. Furthermore, we show that this apparent functional inactivation of Ikaros potentially contributes to T cell imbalance and may have clinical relevance as a similar trend was observed in a translatable, pancreatic cancer mouse model. In conclusion, this study highlights the importance of Ikaros in regulating T cell immune responses in pancreatic cancer hosts.
